# Radiofrequency-Targeted Vertebral Augmentation: Case Report of a Patient with 7 Osteoporotic Vertebral Fractures in a Variant of Osteogenesis Imperfecta

**DOI:** 10.1155/2017/7191476

**Published:** 2017-10-04

**Authors:** Leonard Westermann, Peer Eysel, Marvin Simons, Kourosh Zarghooni

**Affiliations:** Center for Orthopedic and Trauma Surgery, University Medical Center, Kerpener Str. 62, 50937 Cologne, Germany

## Abstract

**Introduction:**

Radiofrequency-targeted vertebral augmentation (RF-TVA) is a recognized treatment for painful compression fractures. RF-TVA in a patient with multiple compression fractures due to type I osteogenesis imperfecta (OI) has not been previously reported.

**Case Presentation:**

A 54-year-old patient with type I OI is presented with a segmental thoracic hyperkyphosis and 7 recent vertebral compression fractures. Because of persistent severe thoracolumbar back pain despite conservative therapy, RF-TVA was indicated. Nocturnal back pain was almost completely relieved at all postoperative time points evaluated. However, overall pain relief dropped only slightly from 7 to 5 on the numerical rating scale (NRS) at the 6-week follow-up, and there was only a small decrease in the Oswestry Disability Index (ODI) from 72% to 63%. An MRI at the 3-month follow-up revealed hyperintensity at levels T11 and T12, indicating slight recollapsing. At the 6-month follow-up, the ODI improved to 55%, although overall pain had worsened to 6 on the NRS. Pain at rest remained at a very low level.

**Conclusion:**

Despite the remaining lumbago, RF-TVA may be a good option for patients with OI who have multiple fractures. However, fractures at multiple levels and segmental thoracic hyperkyphosis may increase the risk for recollapsing and ongoing pain.

## 1. Introduction

Vertebral compression factures are common and painful in patients with osteoporosis and osteogenesis imperfecta (OI). Adequate treatment is required to relieve serious back pain, restore activities of everyday life, and maintain spine alignment in the sagittal profile. Conservative treatment frequently provides only short-term pain relief [1, 2], in which case balloon kyphoplasty is a common procedure when surgery is indicated. Radiofrequency-targeted vertebral augmentation (RF-TVA), developed from the balloon kyphoplasty procedure, is a recent augmentation method that uses high-viscosity cement [3, 4]. Compared to traditional balloon kyphoplasty, RF-TVA offers a significantly shortened operation time with comparable clinical results and equal, or even reduced, cement leakage rates [5–7]. Recent studies have demonstrated that RF-TVA represents a promising alternative approach in the treatment of compression fractures [8].

We extended the indication of RF-TVA to a 54-year-old woman with multiple compression fractures of Th8–12, L1, and L3, along with diminished osseous stability owing to type I osteogenesis imperfecta and osteoporosis. To our knowledge, this is the first case report describing treatment of a multilevel-fractured spine with RF-TVA.

## 2. Case Report

### 2.1. History and Examination

A 54-year-old woman with type I OI, osteoporosis (*T*-Score of −3.3 at the lumbar spine), and at the time untreated Crohn's disease presented at the orthopedic institution with serious thoracolumbar back pain over the previous 5 months and lower back pain over the previous 4 months, without preceding trauma. Several applications of cortisone (in total 3,09 g of prednisolone) over the previous 15 years were used for treating her Crohn's disease.

Because of severe back pain, the patient was not able to walk for more than 500 m and had several limitations in everyday life. She had experienced approximately 25 fractures without external force since the age of 1 year. Further, she reported having a brother, father, and grandmother, each with osteogenesis imperfecta.

Conservative treatment with pain killers and physiotherapy failed. The recommended bisphosphonate therapy was not started due to time issues. Physical examination revealed hyperkyphosis of the thoracic spine without scoliosis. The patient had throbbing pain of the thoracolumbar spine, and paravertebral myogelosis was observed. There was no sign of spinal cord compression or cauda equine syndrome. The patient rated her back pain as 7/10 on the numerical rating scale (NRS). When lying, the pain worsened to 10/10. The Oswestry Disability Index (ODI) was 72%, constituting a crippling condition. The ODI was determined in German [9].

**Figure 1:**
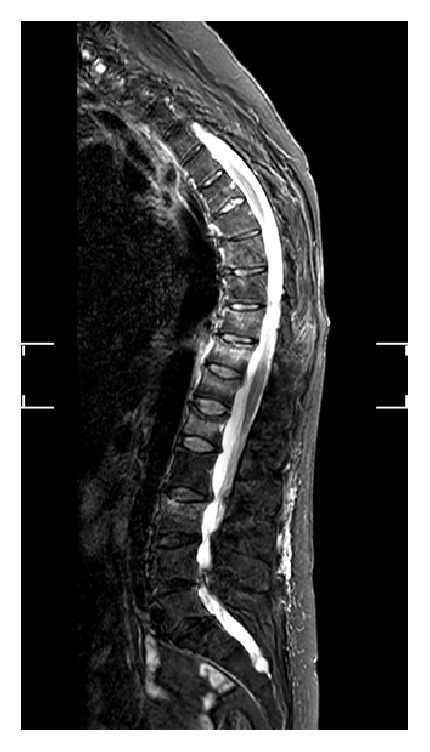
Preoperative sagittal magnetic resonance image (STIR sequence) of the lumbar spine showing edema of multiple vertebral bodies.

Radiographs showed multiple compression fractures in the thoracic and lumbar regions, and high signal on the STIR sequence of an MRI was observed at levels Th8–12, L1, and L3 (see also Figure 1). An additional older compression fracture was present at L4 without any enhanced signal. The patient subsequently underwent RF-TVA.

Regarding the sagittal profile, X-ray of the whole vertebrae showed a thoracic hyperkyphosis of 70 degrees and a C7 offset of 4.3 cm.

### 2.2. Operation

The patient was placed in a prone position on a radiolucent operating table. The compression fractures were localized fluoroscopically using a conventional C-arm device (Endura/Philips/Netherlands). Under fluoroscopic guidance, an introducer was inserted through a small skin incision into either the right or the left pedicle at levels T8–T12 and L3. Access was bipedicular only at level L1. The navigational MidLine Osteotome (DFINE) was inserted through the introducer and guided fluoroscopically. After creating a three-dimensional cavity in the center of the fractured vertebrae, the radiofrequency-activated cohesive ultrahigh-viscosity PMMA cement was delivered at a controlled rate into the cavity under continuous pressure from the hydraulic assembly. The cement volume per vertebral body ranged from 2.2 to 4.3 mL. The intent was to restore and strengthen the vertebrae by injecting the cement primarily into the anterior part of each vertebral body. The total time from incision to suturing was 100 minutes. The patient tolerated the intervention well without pulmonary or neurological complications.

### 2.3. Postoperative Course

The procedure was conducted during a brief in-hospital stay. Immediately after the treatment, the patient's NRS back pain rating decreased from 7 to 5, allowing some activities of her daily life to be restored. Pain reduction in the lying position was more evident, with a decrease in the NRS rating from 10 to 2. Immediately after surgery, the patient reported being able to sleep at night without pain interruptions. At the 6-week follow-up, the ODI had decreased from 72% to 63%.

**Figure 2:**
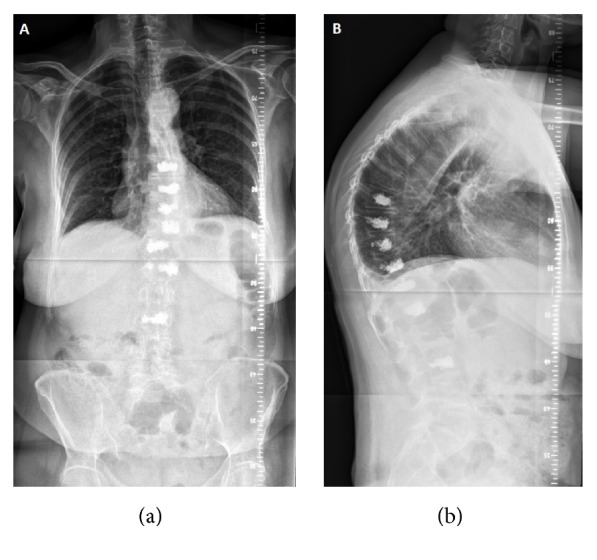
Postoperative anteroposterior (a) and lateral (b) radiographs of the spine.

Figure 2 shows postoperative radiographs with integrated PMMA at affected sites.

**Figure 3:**
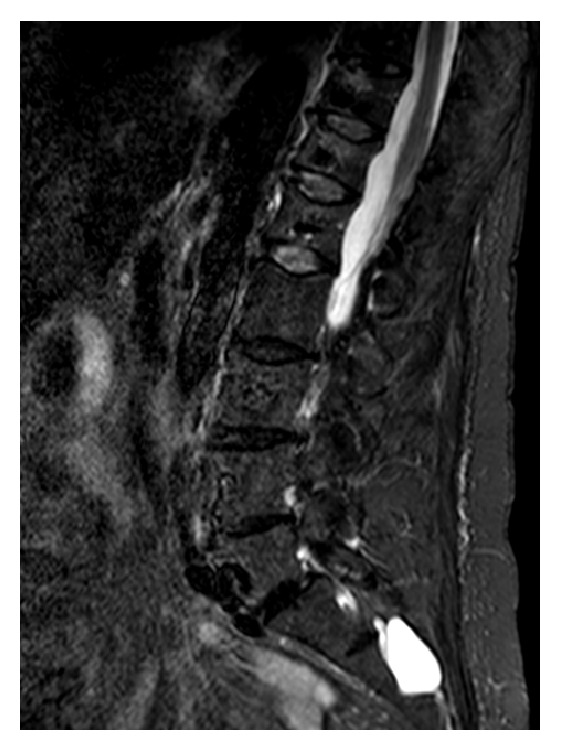
Postoperative sagittal magnetic resonance image of the spine 3 months after surgery, showing increased signal and signs of slight recollapsing, especially in the thoracolumbar transition on the STIR sequence.

Because of ongoing pain 3 months after surgery, we performed another MRI of the lumbar spine. The MRI revealed small hyperintense signals at levels T11 and T12, indicating the possibility of slight recollapsing (see also Figure 3). At the 6-month follow-up, the patient stated that her overall back pain had worsened to a rating of 6 on the NRS, and the patient still was not able to work. However, pain at night remained almost completely relieved. Although the ODI improved to 55%, this still constituted a severe disability.

## 3. Discussion

The patient in this study had type I osteogenesis imperfecta, which is the most benign form of a group of connective tissue disorders that cause skeletal abnormalities such as bone fragility and deformity [10]. Vertebral compression fractures often occur with age or disease processes due to deteriorating bone consistency [11]. In this case, the patient experienced 7 vertebral compression fractures due to low bone density (*T*-Score of −3.3) associated with OI and osteoporosis secondary to long-lasting cortisone treatment for Crohn's disease.

Corporal disability and severe lumbago unresponsive to conservative therapy indicated that surgical intervention was appropriate for the patient. Several operative procedures were available for treatment, but the large number of fractures and very low bone quality limited the practical options. Standard approaches would have required extensive instrumentation, with high risks of screw loosening, establishing bone union, and perioperative complications. Under these circumstances, we considered vertebroplasty and kyphoplasty as possible alternatives before selecting RF-TVA. This treatment had several novel characteristics and improvements over standard therapy. In particular, the operating time in RF-TVA was shortened because the unipedicular approach is often sufficient to achieve adequate cement distribution [3, 7, 12]. Simultaneously, there is also a reduction in the risk of spinal cord damage with single transpedicular access. While treatment success with RF-TVA is well documented in the literature, there are currently no studies that have evaluated RF-TVA for patients with only OI [6, 7, 13]. The few published case reports dealing with OI used conventional vertebroplasty or kyphoplasty [14, 15].

Similar to balloon kyphoplasty, RF-TVA is associated with some common adverse events. However, both procedures are considered safe and the rates of PMMA extravasation are low [6, 7]. It is possible that published complications in OI cases may be related to increased bone porosity in these patients [16, 17]. We expected that RF-TVA would have an advantage relative to balloon kyphoplasty in minimizing the destruction of remaining native spongiosa structures during balloon expansion [18]. This relatively gentle approach could be even more important in similar cases with both OI and severe osteoporosis. It may be possible to preserve more intact cancellous bone while using a navigational osteotome to create a desired transpedicular path via only a single pedicle.

Although the patient reported noticeable pain relief, she remained disabled because of ongoing severe lumbago and was consequently not able to work. Nevertheless, the patient appreciated the pain relief, which enabled her to sleep continuously and improved her quality of life. Her stagnating improvement at the 6-month follow-up may have been related to the disease severity and the presence of fractures across 7 vertebral levels. Most published studies have evaluated patients with only up to 3-level fractures. The patient did not have an adjacent segment fracture, though she had a segmental thoracic hyperkyphosis, which might be a risk factor for new fractures at adjacent levels [19]. Adjacent fractures are a frequent complication after kyphoplasty with an incidence rate of 3–29% [20].

Another reason for ongoing pain may have been the application of an insufficient cement volume in some of the vertebrae. This could have resulted in the enhanced signals seen on the postoperative magnetic resonance image 3 months after surgery, indicating a process of recollapsing without significant secondary loss of height restoration. There is no recommendation in the literature for a specific amount of cement to achieve optimal results, although a biomechanical study by Liebschner et al. suggests that 3.5 mL in L1 is necessary to adequately restore stiffness [21]. However, other authors suggest that larger volumes are necessary [22, 23]. Clinical data show that smaller volumes of cement (2.9 mL in thoracic vertebrae and 3.0 mL in lumbar vertebrae) may be sufficient to reduce pain and restore vertebral height [24]. A recent review by Papanastassiou et al. reported that there is growing evidence of a correlation between larger cement volumes and both better restoration of sagittal alignment and improved pain resolution [24–28]. The mean volume of cement used in the different studies (ranging from 3.4 to 4.0 mL) was probably slightly higher in comparison to this study [6].

RT-TVA should be considered as a potential treatment for patients with OI who have numerous vertebral fractures and for whom conservative treatment has been ineffective. We believe that it is probably the most suitable surgical procedure in such cases, even though the pain relief observed in this study was not comparable to what may be expected for patients with 1-level or 2-level fractures. The patient reported that the initial pain relief at night immediately after RF-TVA was of sufficient value that in hindsight she would elect to undergo the treatment again.

## 4. Conclusion

RF-TVA is a minimally invasive therapeutic procedure offering an alternative to standard treatments for multiple vertebral fractures in patients with type I osteogenesis imperfecta and pain that is unresponsive to conservative treatment. In this case report, the patient improved only slightly in overall ODI and pain level, but this may have been because of the multilevel fractures or segmental kyphosis. The patient did, however, experience immediate and significant pain relief at night, which led her to report that the procedure was worthwhile.
